# Coexistence of pyoderma gangrenosum and sweet's syndrome in a patient with ulcerative colitis

**DOI:** 10.11604/pamj.2015.21.151.6364

**Published:** 2015-06-24

**Authors:** Faida Ajili, Asmahan Souissi, Fathi Bougrine, Najah Boussetta, Nadia Ben Abdelhafidh, Sameh Sayhi, Bassem Louzir, Nejib Doss, Janet Laabidi, Salah Othmani

**Affiliations:** 1 Department of Internal Medicine, Military Hospital of Tunis, 1008 Montleury, Tunisie; 2 Department of Dermatology, Military Hospital of Tunis, 1008 Montleury, Tunisie; 3 Department of Anatomopathology, Military Hospital of Tunis, 1008 Montleury, Tunisie

**Keywords:** Pyoderma gangrenosum, sweet′s syndrome, neutrophilic dermatosis, ulcerative colitis

## Abstract

Pyoderma gangrenosum (PG) and Sweet's Syndrome (SS) are inflammatory skin diseases caused by the accumulation of neutrophils in the skin and, rarely, in internal organs. These neutrophilic dermatosis (NDs) are distinguished by the existence of forms of transition or overlap. They are frequently associated to systemic diseases especially hematologic and gastrointestinal ones. We report a case of a patient with ulcerative colitis (UC) who successively developed two types of NDs: PG then SS. A 66 years old patient with a history of UC consulted in July 2012 for an erythematous swelling of the back of the right hand treated with antibiotics without improvement. At that time, bacteriological samples were negative. In October 2012, he was hospitalized for polyarthralgia and impaired general condition. In physical examination, he had vesiculobullous plaque of 10 cm long of the right hand and wrist, infiltrated erythematous plaque on the right leg and another topped with a large pustule at the left ankle. Skin biopsy showed at the back of the right hand an aspect of PG and at the infiltrated plaques of the ankle an aspect of SS. Prednisone was started with improvement of the skin lesions and a recovery condition. The combination of PG and SS has already been described in cases of hematologic malignancy and rarely in UC. There is also the notion of passage from a neutrophilic dermatosis to another. Indeed, a typical lesion initially of SS can evolve to a future PG. This case demonstrates that neutrophilic dermatoses form a continous spectrum of entities that may occur in UC.

## Introduction

Neutrophilic dermatoses (NDs) are characterized histologically by an epidermal and/or dermal infiltrate of polymorphonuclear leucocytes, absence of microorganisms on special stains and culture. This group of diseases includes, Sweet′s syndrome (SS), pyoderma gangrenosum (PG), erythema elevatum diutinum, neutrophilic eccrine hidradenitis and Sneddon Wilkinson disease [1]. Recently, NDs have been included among the autoinflammatory diseases, which are due to mutations of genes regulating the innate immune responses [2]. These entities are distinguished by the existence of forms of transition or overlap and the frequency of their association with systemic diseases especially hematologic and gastrointestinal ones. We report a case of a patient with ulcerative colitis (UC) who successively developed two types of NDs: PG then SS.

## Patient and observation

A 66 year old patient with a history of UC treated by mesalazine since 5 years consulted in July 2012 for a painful erythematous swelling of the back of the right hand. The bacteriological samples were negative, the lesion was considered as an abscesses and the patient was treated with antibiotics and local antiseptics without improvement. The evolution was characterized by the appearance of an erythematous vesiculobullous centrifugal expansion taking the whole back of the right hand treated several times by various antibiotics unsuccessfully. Biopsy of this lesion was initially not specific. In October 2012, the patient was hospitalized for fever and polyarthralgia in the context of impaired general condition. He had a quiescent UC disease.

**Figure 1:**
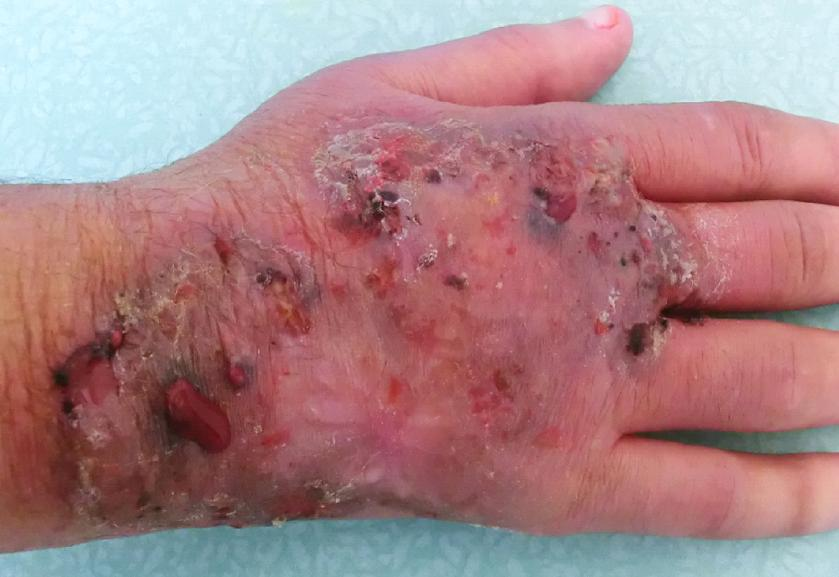
Erythematous plaque with vesicular bubbles, elevated edge, interesting the back of the right hand and wrist

**Figure 2:**
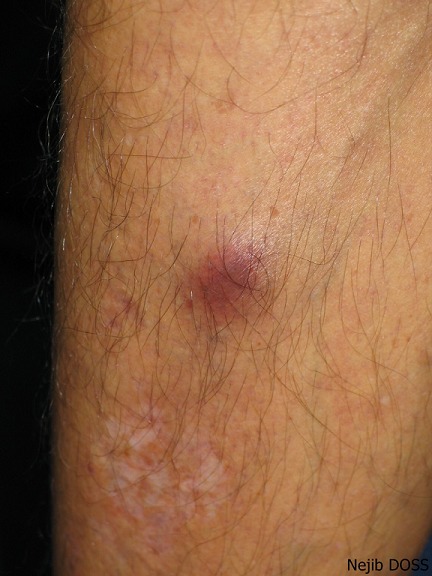
An infiltrated erythematous plaque on the right leg

**Figure 3:**
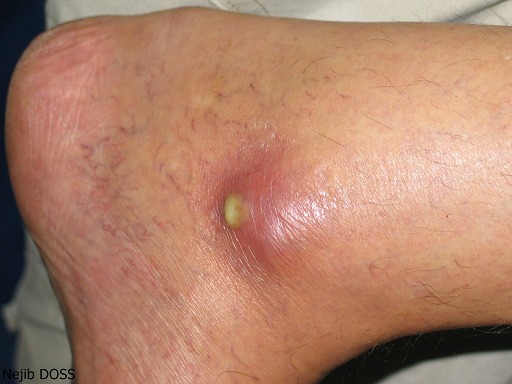
Infiltrated erythematous plaque of the left ankle surmounted by a large pustule

**Figure 4:**
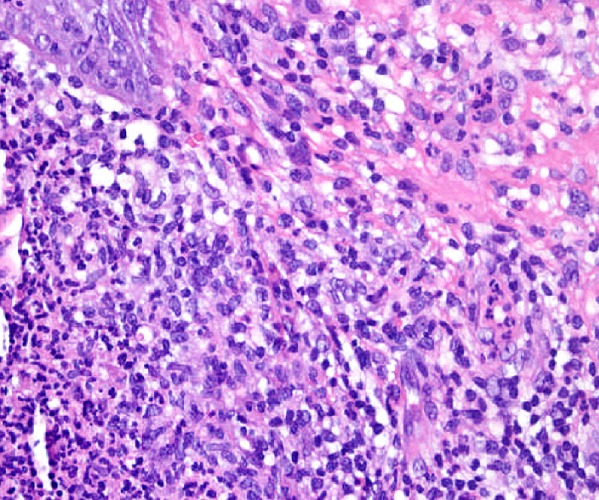
Pyoderma gangrenosum: dermal polynuclear suppuration with vasculitis: (HE x 200)

**Figure 5:**
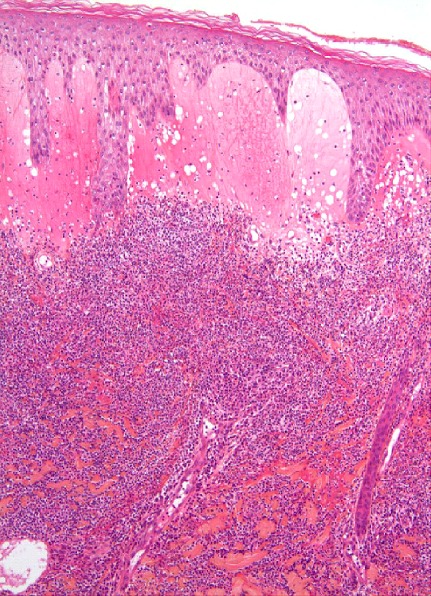
Papillary dermal edema and intense dermal neutrophilic infiltrate with leucocytoclasia without vasculitis

**Figure 6:**
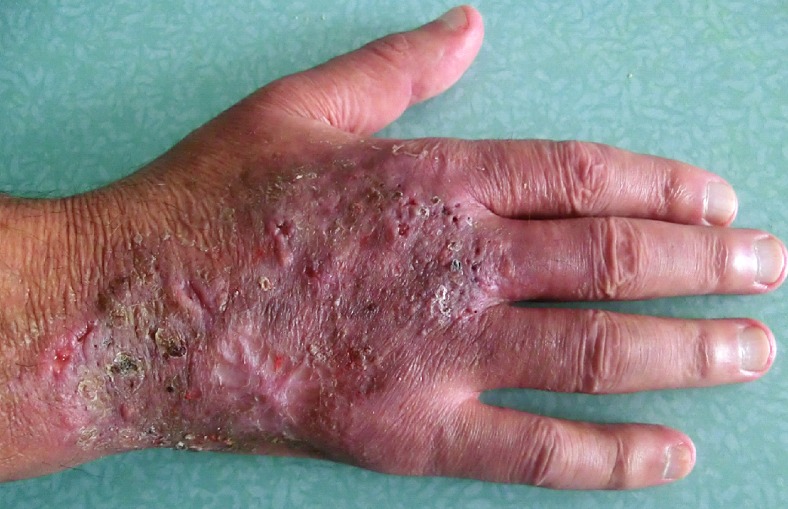
Early desinfiltration and healing in day 7 of treatment with regression of bubbles

The skin examination found an erythematous plaque of 10 cm of diameter, with a raised border and vesicules, taking the back of the right hand and wrist (Figure 1). There was also an infiltrated erythematous plaque on the right leg (Figure 2) and another topped with a large pustule at the left ankle (Figure 3). In biology, there was a biological inflammatory syndrome and high leukocytosis with neutrophils. Hepatic and renal functions were normal. Skin biopsy showed at the edge of the back of the closet right hand ulcerated epidermis and the dermis infiltrate rich in neutrophils with leukocytoclastic vasculitis finding a PG (Figure 4). Other biopsies taken at infiltrated erythematous plaques of the ankle showed a normal appearance of skin, edema of the superficial dermis based on an infiltrate rich in neutrophils without vasculitis confirming the diagnosis of SS (Figure 5). Corticosteroid treatment was then started with prednisolone at a dose of 1mg/Kg/j. The evolution was marked since day 7 of treatment by the desinfiltration of the plaques (Figure 6), the recovery of the general condition and disappearance of biological inflammatory syndrome.

## Discussion

SS and PG are the most common neutrophilic dermatosis. They have many features in common: neutrophilic infiltrate, altered immunologic reactivity, response to immunosuppressives and reports of coexistence in a single patient [3]. The combination of these two entities has already been described in cases of hematologic and much more rarely in the case of chronic inflammatory bowel disease [4]. In fact, there is an overlap between the two conditions with lesions categorised as SS actually being clinically more characteristic of atypical bullous PG and vice versa [3]. Indeed, a typical lesion initially of SS can evolve to a future PG.

Other associations of NDs have been reported: PG and subcorneal pustular dermatosis [5], PG and aseptic spleen abscess [6]. We are actually dealing with a "neutrophilic disease". Our observation showed that neutrophilic dermatoses are a continous spectrum as our patient developed first PG followed by SS. Unlike PG, the incidence of which, in UC patients, is about 1-3% [2], SS is very rare. The increasing number of patients affected by intestinal bowel diseases (IBD) developing SS during the last years, together with the evidence that cutaneous manifestations of IBD are very frequent, would appear to suggest that there is a relationship, possibly a common pathogenetic mechanism, between IBD and SS. Although NDs have been described also in some patients with quiescent IBD, the occurrence of the disorder has frequently been reported during relapse of disease. The patient described here had a quiescent disease when the NDs had occurred.

In histological samples, the lack of vasculitis and the abrupt onset of dermatosis without ulcerations allow the SS to be dinguished from PG. Malone et al suggested that vasculitis may be present in some patients with Sweet's syndrome. It is than an epiphenomen due to the release of noxious products from neutrophils, especially in the cases of lesions of long duration [7]. In our patient, histology was typical for PG and SS without vasculitis in the second one.

Concerning the treatment of NDs, usually four to six weeks of glucocorticoid therapy are needed [2]. Agents that have been successfully implemented as glucocorticoid-sparing agents include dapsone, azathioprine [8, 9], tacrolimus, mycophenolate mofetil, methotrexate, chlorambucil, and clofazimine [2]. Tumor necrosis factor-α inhibitors, such as adalimumab or infliximab, have been shown to be helpful with refractory disease, especially in patients with inflammatory bowel disease [10]. SS and PG are usually responsive to oral corticosteroids, and the immunosuppressant should be considered in resistant or highly relapsing cases. In our case, the UC was quiescent so our patient was treated with corticoids alone with a good clinical and biological evolution.

## Conclusion

PG and SS are distinguished by the existence of forms of transition or overlap. They are frequently associated to systemic diseases especially hematologic and gastrointestinal ones. Our observation demonstrated that neutrophilic dermatoses form a continous spectrum of entities that may occur in UC.
